# Multivariate GWAS analysis reveals loci associated with liver functions in continental African populations

**DOI:** 10.1371/journal.pone.0280344

**Published:** 2023-02-21

**Authors:** Chisom Soremekun, Tafadzwa Machipisa, Opeyemi Soremekun, Fraser Pirie, Nashiru Oyekanmi, Ayesha A. Motala, Tinashe Chikowore, Segun Fatumo

**Affiliations:** 1 The African Computational Genomics (TACG) Research Group, MRC/UVRI, and LSHTM, Entebbe, Uganda; 2 Department of Immunology and Molecular Biology, College of Health Science, Makerere University, Kampala, Uganda; 3 H3Africa Bioinformatics Network (H3ABioNet) Node, Centre for Genomics Research and Innovation, NABDA/FMST, Abuja, Nigeria; 4 Department of Medicine, University of Cape Town and Groote Schuur Hospital, Cape Town, South Africa; 5 Department of Medicine, Hatter Institute for Cardiovascular Diseases Research in Africa and Cape Heart Institute, University of Cape Town, Cape Town, South Africa; 6 Population Health Research Institute, David Braley Cardiac, Vascular and Stroke Research Institute, Hamilton, Ontario, Canada; 7 Thrombosis and Atherosclerosis Research Institute, David Braley Cardiac, Vascular and Stroke Research Institute, Hamilton, Ontario, Canada; 8 Department of Pathology and Molecular Medicine, McMaster University, Michael G. DeGroote School of Medicine, Hamilton, Ontario, Canada; 9 Molecular Bio-Computation and Drug Design Laboratory, School of Health Sciences, University of KwaZulu-Natal, Westville Campus, Durban, South Africa; 10 Department of Diabetes and Endocrinology, University of KwaZulu-Natal, Durban, South Africa; 11 Sydney Brenner Institute for Molecular Bioscience, Faculty of Health Sciences, University of the Witwatersrand, Johannesburg, South Africa; 12 Department of Pediatrics, MRC/Wits Developmental Pathways for Health Research Unit, Faculty of Health Sciences, University of the Witwatersrand, Johannesburg, South Africa; 13 Department of Non-Communicable Disease Epidemiology, London School of Hygiene and Tropical Medicine, London, United Kingdom; Children’s hospital of philadelphia, UNITED STATES

## Abstract

**Background:**

Liver disease is any condition that causes liver damage and inflammation and may likely affect the function of the liver. Vital biochemical screening tools that can be used to evaluate the health of the liver and help diagnose, prevent, monitor, and control the development of liver disease are known as liver function tests (LFT). LFTs are performed to estimate the level of liver biomarkers in the blood. Several factors are associated with differences in concentration levels of LFTs in individuals, such as genetic and environmental factors. The aim of our study was to identify genetic loci associated with liver biomarker levels with a shared genetic basis in continental Africans, using a multivariate genome-wide association study (GWAS) approach.

**Methods:**

We used two distinct African populations, the Ugandan Genome Resource (UGR = 6,407) and South African Zulu cohort (SZC = 2,598). The six LFTs used in our analysis were: aspartate transaminase (AST), alanine transaminase (ALT), alkaline phosphatase (ALP), gamma-glutamyl transferase (GGT), total bilirubin, and albumin. A multivariate GWAS of LFTs was conducted using the exact linear mixed model (mvLMM) approach implemented in GEMMA and the resulting P-values were presented in Manhattan and quantile-quantile (QQ) plots. First, we attempted to replicate the findings of the UGR cohort in SZC. Secondly, given that the genetic architecture of UGR is different from that of SZC, we further undertook similar analysis in the SZC and discussed the results separately.

**Results:**

A total of 59 SNPs reached genome-wide significance (P = 5x10^-8^) in the UGR cohort and with 13 SNPs successfully replicated in SZC. These included a novel lead SNP near the *RHPN1* locus (lead SNP rs374279268, P-value = 4.79x10^-9^, Effect Allele Frequency (EAF) = 0.989) and a lead SNP at the *RGS11* locus (lead SNP rs148110594, P-value = 2.34x10^-8^, EAF = 0.928). 17 SNPs were significant in the SZC, while all the SNPs fall within a signal on chromosome 2, rs1976391 mapped to *UGT1A* was identified as the lead SNP within this region.

**Conclusions:**

Using multivariate GWAS method improves the power to detect novel genotype-phenotype associations for liver functions not found with the standard univariate GWAS in the same dataset.

## Background

Liver disease is any condition that causes liver damage and inflammation and may likely affect the function of the liver [1]. Globally, liver disease has been found to contribute greatly to the mortality and morbidity rate in the world [2]. In sub-Saharan Africa (SSA), its prevalence, distribution, and aetiology have not been well characterised, but the little that is known is that liver disease causes approximately 200,000 deaths annually, primarily due to liver cirrhosis and hepatocellular carcinoma [3]. Globally, risk factors for liver disease include gender, ethnicity, and socio-economic status [4]. In SSA, more than 80% of the burden of liver disease has been attributed to endemic blood borne virus (BBV) infections, alcohol, hepatotoxic medications (including traditional and herbal medicines), non-alcoholic fatty liver disease (NAFLD), and exposure to aflatoxins [5–7]. To improve screening for liver disease, and to implement appropriate investigations and intervention, liver biomarker levels in continental Africans need to be better understood.

Liver function tests (LFTs) are vital screening tools for liver biomarker levels that can be used to determine the health of the liver, detect hepatic dysfunction, and predict timely intervention for liver diseases [8]. LFTs are biochemical tests performed by accessing the levels of total proteins, albumin, bilirubin, and liver enzymes in the blood [9]. The most commonly measured liver biomarkers include alanine aminotransferase (ALT) and aspartate aminotransferase (AST), which are markers of hepatocyte injury; alkaline phosphatase (ALP), which can be a marker of biliary disease and cholestasis; bilirubin, gamma-glutamyl transferase (GGT) and albumin which are marker of parenchymal liver disease or biliary obstruction [10].

Several factors are associated with the liver biomarker levels in individuals such as genetic and environmental factors. For instance, variation in the liver enzyme levels in humans was found to be moderately heritable, 22–60% [11]. Although the genetic variation varies across individuals and genetics may not completely explain this heritability, genetics could however be used in stratifying these individuals based on their polygenic risk score on who may develop liver disease over a period and identify individuals who will benefit from an intervention [11]. A good example is patatin-like phospholipase domain-containing protein 3 (*PNPLA3*) genetic testing for NAFLD susceptibility and hereditary hepatic disease, which is used clinically in Europe and the US. The first evidence of *PNPLA3* being associated with NAFLD was revealed by a genome-wide association study (GWAS) of Hispanic, African American, and European American individuals in the Dallas Heart Study back in 2008 [12]. Currently, *PNPLA3* is undergoing testing as a promising therapeutic target for personalized treatment of chronic liver disease [13].

GWASs are used in detecting associations between common genetic variants and disease/phenotypic traits in sampled populations [14]. Multivariate GWAS methods involve the joint analysis of potentially correlated traits and are known to increase the statistical power to detect associations in the case of shared genetic basis between traits [15, 16]. Previous study has shown that. liver enzymes are correlated, for example, chen et al., found that the phenotypic Pearson correlation between ALT and AST was 0.67, and 0.19 between either ALT and ALP or AST and ALP. They further highlighted that the co-heritability between ALT and AST was 0.67, while that between ALT and ALP was 0.24 and between AST and ALP 0.21 [17].

Interestingly, genetic determinants of disease vary significantly between populations, with the highest diversity in the cradle of humankind, Africa. Hence, from the ‘Out-of-Africa’ theory, the inclusion of populations of continental African ancestry is crucial. In genetic studies, including continental African ancestry expands the breadth of novel findings from genetic variation, migration, to the etiology of complex diseases, on the continent and globally. This is due to the smaller linkage disequilibrium (LD) blocks in Africans, the genetic heterogeneity found in African populations and recently, the over 3 million new variants discovered in an African genome project [16, 18]. In our previous study [19], we explored the utility of univariate analysis in understanding the genetic drivers of liver biomarkers, however univariate analysis limited the potential of discovery. Thus, the goal of our study was to expand our knowledge on the genetic architecture of liver biomarkers by identifying genetic loci associated with liver biomarker levels with a shared genetic basis in continental Africans, using a multivariate GWAS approach.

## Methods

### Study population

We used two genetically distinct African populations, the Ugandan Genome Resource (UGR = 6,407) and South Africa Zulu cohort (SZC = 2,598) [19, 20]. The UGR is a genotyped dataset from the Uganda General Population Cohort (GPC). The GPC started its round 22 study in 2011, to investigate the genetics and epidemiology of communicable and non-communicable diseases in children and adults [21]. Subsequently, the SZC consists of an amalgam of both the Durban Diabetes Study and the Durban Diabetes Case Control study. DDS is a population-based cross-sectional study, established to allow researchers to gain insight into the population-based prevalence of type 2 diabetes (T2D) and its associated risk factors among African descendant resident in the city of Durban, South Africa. DCC is a study of individuals of Zulu descent, resident in KwaZulu-Natal, aged > 40 years and with a diagnosis of T2D (based on the WHO criteria).

The UGR GPC study was approved by Uganda Virus Research Institute Science and Ethics Committee and the Uganda National Council for Science and Technology (UNCST). The Biomedical Research Ethics Committee at the University of KwaZulu-Natal (reference: BF030/12) and the UK National Research Ethics Service (reference: 14/WM/1061) approved the DDS study. The DCC study was approved by the UK National Research Ethics Service (reference: 11/H0305/6) and the Biomedical Research Ethics Committee at the University of KwaZulu-Natal (reference: BF078/08). Sample and biochemical processes have been described in [19, 20].

### Biochemical and genetic samples collected

#### Genotyping and quality control (QC)

The UGR is a combination of 5,000 genotyped UGWAS and 2,000 sequenced samples. The final quality controlled UGR data include 6,407 individuals (4,429 genotyped data and 1,978 sequenced data). The UGR DNA samples were genotyped on the Illumina HumanOmni 2.5M BeadChip array at the Wellcome Trust Sanger Institute (WTSI). The UGR samples underwent whole-genome sequencing with an average coverage of 4x for each sample on the Illumina HiSeq 2000 with 75bp paired end reads. The SZC which consist of DDS (n = 1,165) and DCC (n = 1,542) had a total sample size of 2,707 samples. However, the final quality-controlled data include 2,598 individuals. DNA samples were genotyped on the consortium-driven Illumina HumanOmni MultiEthnic GWAS/Exome Array (MEGA pre-commercial v1) using the Infinium Assay. Detailed quality control and imputation information has been previously reported by [19, 22].

### Statistical analysis

A multivariate GWAS of six liver biomarkers i.e. ALT, AST, ALP, bilirubin, GGT and albumin were conducted using the exact linear mixed model approach implemented in GEMMA, mvLMM [23]. We adjusted for age, age squared, and sex for both the UGR and SZC. We plotted the resulting P-values from this association analysis and visualized using Manhattan and quantile-quantile (QQ) plots. A genome-wide significance (GWS) threshold of p<5×10^−8^ was applied and p<0.05 for the SZC. The multivariate linear mixed model (mvLMM) implemented in GEMMA accounts for population structure and genetic background by estimating the genotype effect as a variable in a linear mixed model by explicitly incorporating a similarity matrix known as the genomic relationship matrix (GRM) between the individuals.

#### Downstream in-silico functional analysis

Since single marker approach commonly used in most GWAS do not pick up candidate genes with attenuated effects, and the direct functional analysis using humans, cells lines or any study model that proxies human is expensive. Gene and pathway enriched methodologies have been developed to circumvent these limitations [24, 25]. Approaches like this also provide the opportunity to increase discovery by aggregating the joint effects of weakly associated SNPs [26]. Functional information from biological databases such as Genotype-Tissue Expression |(GTEx) [27], Encyclopedia of DNA Elements (ENCODE) [28], Roadmap Epigenomics project [29] and Chromatin interaction information [30] are all integrated to identify and prioritize candidate genes. Regional association plots were generated using LocusZoom [31]. We investigated lead associations for novelty using the GWAS Catalog (https://www.ebi.ac.uk/gwas/), GeneAtlas Phenome-Wide Association Studies (PheWAS- http://geneatlas.roslin.ed.ac.uk/phewas/), GWAS Atlas PheWAS (https://atlas.ctglab.nl/PheWAS), the Common Metabolic Diseases Knowledge Portal (CMDKP; https://hugeamp.org/ and the Open Targets Genetics portal (https://genetics.opentargets.org/.

The CMDKP is an open-source platform that is developed to facilitate research in type 2 diabetes, type 1 diabetes, cardiovascular diseases, cerebrovascular diseases, sleep and circadian disorders, including up to 300 traits. The Open Targets is a platform developed to identify and prioritize biological targets in a systematic and functional way. Open Targets integrate data from sources such as GWAS catalogue, European Variation Archive, UniProt, ChEMBL, Expression Atlas, reactome etc. We adjusted the GeneAtlas PheWAS *P* values for false-discovery rates (FDRs) using the Benjamini-Hochberg procedure and mapped the GWS SNPs. We also interrogated the Genotype-Tissue Expression Project (GTEx) portal (https://gtexportal.org/home/) for expression quantitative trait loci (eQTLs) [32]. Functional analyses were conducted using Functional Mapping and Annotation of Genome-Wide Association Studies (FUMA; https://fuma.ctglab.nl/) [33] which uses Multi-marker Analysis of GenoMic Annotation (MAGMA) [24] for gene set analysis. We also performed gene and gene set analyses, while correcting for type 1 error rate and LD, using all the GWAS SNPs.

## Results

### Study population

The UGR cohort consist of 6,407 samples and the SZC has 2,598 samples. For the UGR data, the mean subject age was 34.1 ± 18.3 years, with the minimum age of 13 years and a maximum age of 97 years. Sex-wise, 57.1% of the population were female, while 42.9% were male. The mean subject age for SZC was 49.1 ± 15.3 years, with the minimum age of 18 years and a maximum age of 91 years. The characteristics of the study population are described in Table 1.

**Table 1:** Descriptive characteristics for the study population.

	UGR	SZC
	(N = 6407)	(N = 2598)
Age (years)	34.1 ± 18.3	49.1 ± 15.3
Male, n (%)	2747 (42.9)	679 (26.1)
Female, n (%)	3660 (57.1)	1919 (73.9)
Alanine aminotransferase, ALT(U/L)	20.0 ± 12.3	11.15 ± 8.2
Aspartate aminotransferase, AST (U/L)	27.8 ± 22.3	16.3 ± 11.2
Alkaline phosphatase, ALP (IU/L)	132.7 ±102.1	78.6 ± 31.2
Total Bilirubin (μmol/L)	9.8 ± 8.4	5.2 ± 3.6
Gamma-glutamyl transferase, GGT (U/L)	28.8 ± 66.6	44.1 ± 55.95
Albumin (g/L)	41.5 ± 4.0	38.9 ± 5.0

Data are shown as Mean ± SD

Abnormal LFTs, according to American Reference Range, ARR, are defined as test results outside of the following ranges: ALT (male: 10–55 U/L, female: 7–30 U/L), AST (male: 10–40 U/L, female: 9–32 U/L), GGT (male: 8–61 U/L, female: 5–36 U/L), Total bilirubin (1.71 to 20.5 μmol/L), albumin (35-55g/L) and ALP (male: 45–115 IU/L, female: 30–100 IU/L).

### The multivariate GWAS of LFTs

**Fig 1:**
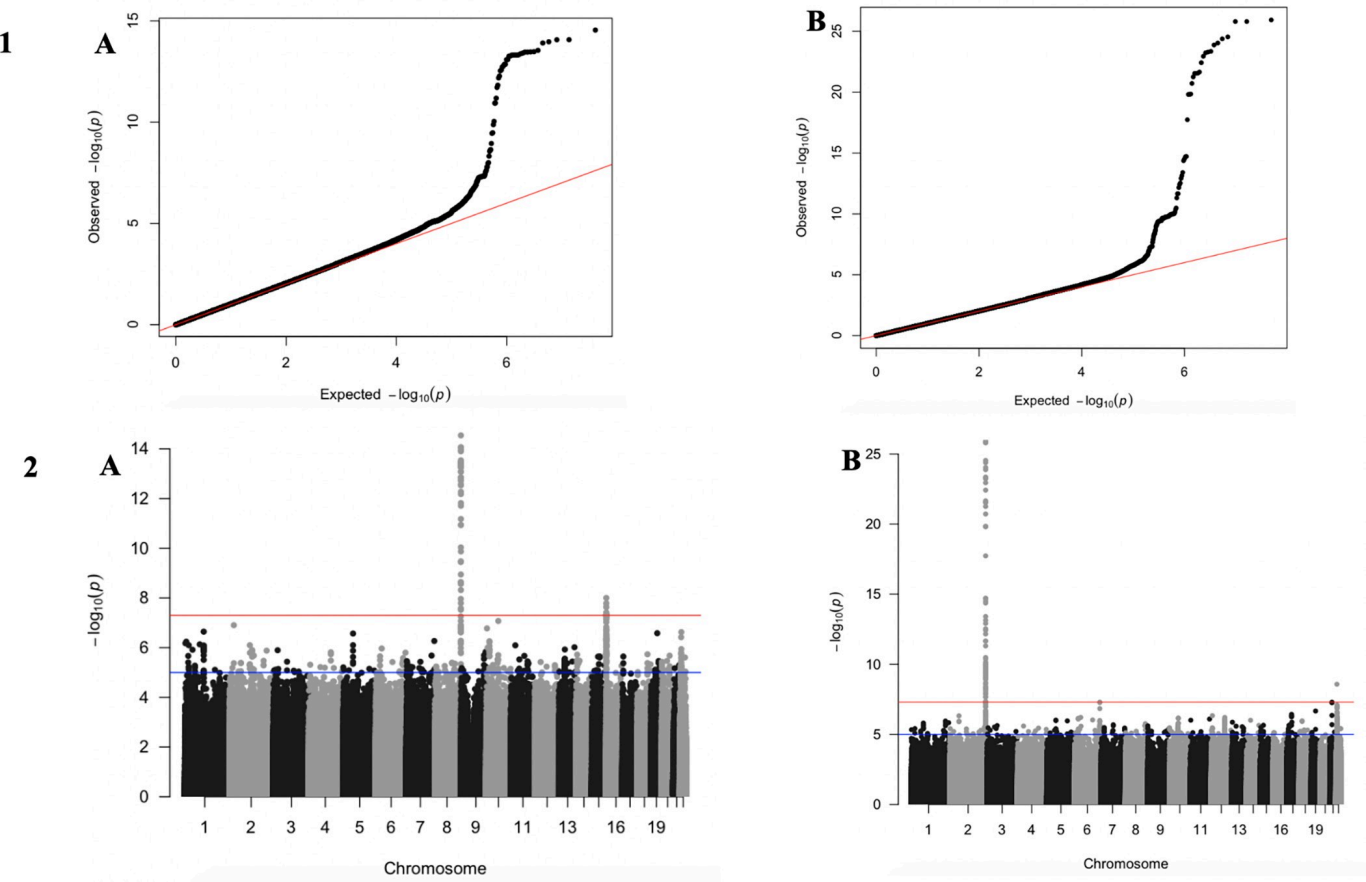
**i**- Quantile-quantile (QQ) plots of the UGRcohort (A) and the SZC cohort (B). The x-axis indicates the negative log-scale of expected p-values of each SNP, while the y-axis represents the negative log-scale of the observed p-values. The straight line indicates the expected results under Hardy-Weinberg equilibrium. The genomic inflation factors for the UGR and SZC cohort were 1.017 and 1.043, respectively **ii:** Manhattan plots showing SNPs associated with liver biomarkers in the UGR cohort (A) and the SZC cohort (B). In the UGR set of the GWAS, 59 SNPs were significantly associated with the correlated liver biomarkers. We performed a validation test by checking how many of the significant SNPs were replicated in the SZC at a threshold of *P* < 5x10^-2^, 13 remained significant after this test.

Autosomal SNPs were tested via multivariate association testing. The plotted QQ plots (Fig 1iA and 1iB), show that several SNPs were associated with correlated liver biomarker levels at a level of GWS, *P* <5 x 10^−8^.

The Manhattan plots (Fig 1iiA and 1iiB) show the SNPs that were associated with liver biomarker levels with a shared genetic basis in (A) UGR and (B) SZC. In UGR, a total of 59 SNPs in chromosome 8 and 16 reflect significant associations with the liver biomarker levels with a shared genetic basis (S1 Table in S1 File). However, in the SZC, only 13 of these SNPs remained significant at a threshold of *P* < 5x10^-2^. The 13 replicated SNPs were: rs77797981, rs115999457, rs114645427, rs116582094, rs78831518, rs141858135, rs143942584, rs146729198, rs116830408, rs114437003, rs116686058, rs148110594 and rs374279268 (S3 Table in S1 File). The effect estimates of these SNPs can be found in the S5 Table in S1 File for UGR and S6 Table in S1 File for SZC. In the mvLMM of SZC, 107 SNPs in chromosome 2 and 22 were significantly associated with the liver biomarker levels (S2 Table in S1 File), however 105 of the SNPs are in linkage disequilibrium with each other and none of the remaining 2 SNPs were replicated in SZC. We further highlighted the previously reported univariate results (Gurdasani et al.) of these two SNPs (S7 Table in S1 File) The closest gene to the SNP at chromosome 22 (rs4148325) is UGT1A (S8 Table in S1 File), while the second SNP has no known gene associated with it. In the SZC, 17 SNPs were significant in the SZC, while all the SNPs fall within a signal on chromosome 2, rs1976391 mapped to *UGT1A* was identified as the lead SNP within this region.

#### Downstream in-silico functional analysis

In order to identify independent and lead SNP with each locus, we undertook a distance clumping of 1 MB (500kb upstream and 500kb downstream) of the lead SNPs using an in-house script, and only two out of the 13 SNPs were left—rs374279268 (4.79 x10^-9^) and rs148110594 (2.34 x10^-8^). This implies that all the other eleven SNPs are in LD with chr 16 lead SNP (Fig 1). The nearest genes to the lead loci were Rhophilin Rho GTPase Binding Protein 1 *(RHPN1)* and Regulator of G Protein Signaling 11 *(RGS11)*, respectively (Table 2; S4 Table in S1 File). S6 Table in S1 File shows the univariate result of the two replicated SNPs.

**Table 2:** Lead SNPs in UGR that replicated in SZC.

						UGR		SZC	
Nearest Gene	rsID	CHR	BP (b37)	EA	NEA	EAF	P-value	EAF	P-value
*RHPN1*	rs374279268	8	144485034	T	C	0.989	4.79x10^-9^	0.984	3.15x10^-2^
*RGS11*	rs148110594	16	321605	T	C	0.928	2.34x10^-8^	0.979	4.07x10^-2^

Abbreviations: rsID (Reference SNP cluster ID), CHR (Chromosome), BP (base position), b37 (the Genome Reference Consortium Human genome build 37) or hg19(Human Genome version 19), EA (effect allele), NEA (non-effect allele), Rhophilin Rho GTPase Binding Protein 1 *(RHPN1)* and Regulator of G Protein Signaling 11 *(RGS11)*.

### Prior findings and previously reported loci for LFTs

In our study, the *RGS11* locus found in the mvLMM UGR cohort replicated (P-value < 0.05) a previously reported finding from a univariate study from Gurdasani *et al*., 2019, as well as in our mvLMM SZC (Table 2). On the other hand, the novel *RHPN1* locus has never been reported to be previously associated with any LFTs traits. Furthermore, we looked up rs374279268 in GTEx and no known eQTLs were found. Similarly, the low MAF of rs148110594 may explain reason no eQTLs was also found. rs1976391 found in the SZC has shown to be associated with metabolite measurement [34], biliverdin measurement [35] and hormone measurement [36] and bilirubin level [37].

**Fig 2:**
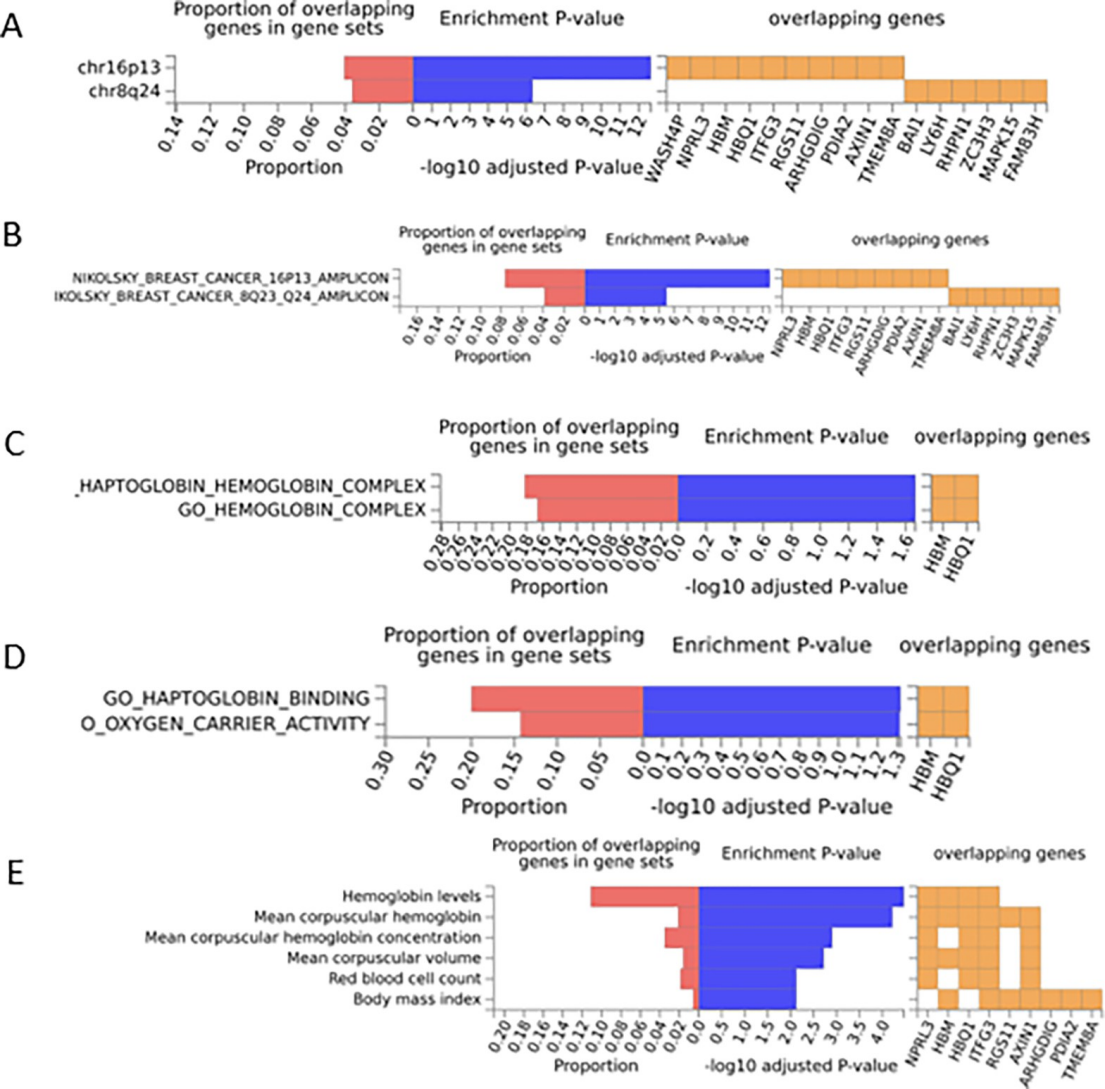
Enrichment of input genes in gene sets for the UGR cohort (A) Positional gene sets (MsigDB c1) (B) Curated gene sets, (C) GO cellular components (MsigDB c5), (D) GO molecular functions (MsigDB c5), (E) GWAS catalog reported genes.

In FUMA, we performed MAGMA analyses, including gene and generalized gene set analyses for our multivariate GWAS data (Table 3A, and Fig 2). This was done by uploading our multivariate summary statistics onto the FUMA website. FUMA is used in the interactive visualisation, functional annotation and gene prioritisation of summary level data. While MAGMA is a tool used for gene and gene-set analysis. We observed significant associations (P-value  < 0 .05 and P_Bonferroni corrected_ < 0.05) with genes and gene sets of relevance, as illustrated in Table 3A. Gene-set analysis gives insight into the association of SNPs at the gene level with biological pathways. The gene sets were obtained by computing the gene set *P- value* using the gene-based *P-value* of 4728 curated genes and 6166 gene ontology terms derived from the MsigDB database [38].

**Table 3:** MAGMA gene set analysis.

Gene Set	N Genes	Beta	Beta STD	SE	P-value	P_Bonferroni_
**A. UGR**						
Curated gene sets: Nikolsky breast cancer 16p13 amplicon	118	0.73	0.08	0.14	1.78x10^-7^	0.003
**B. SZC**						
GO bp:go flavonoid glucuronidation	9	5.3429	0.11535	0.6605	3.18E^-^16	4.93E^-^12
GO bp:go xenobiotic glucuronidation	11	3.2331	0.077165	0.51195	1.38E^-^10	2.13E^-^06

Abbreviations: GO (gene ontology), bp (biological processes) and SE (standard error).

In MAGMA, several gene sets were found to be significant that were known to be associated with Chr16p13, Chr 8q24, breast cancer amplicons, the cellular components of the haptoglobin and hemoglobin complex, and the molecular functions of haptoglobin binding and oxygen carrier activities in Fig 2. We also performed SNP2GENE function analysis in Fig 2. Furthermore, from the SZC, the top gene sets were significantly associated with flavonoid glucuronidation and xenobiotic glucuronidation (Table 3B).

**Fig 3:**
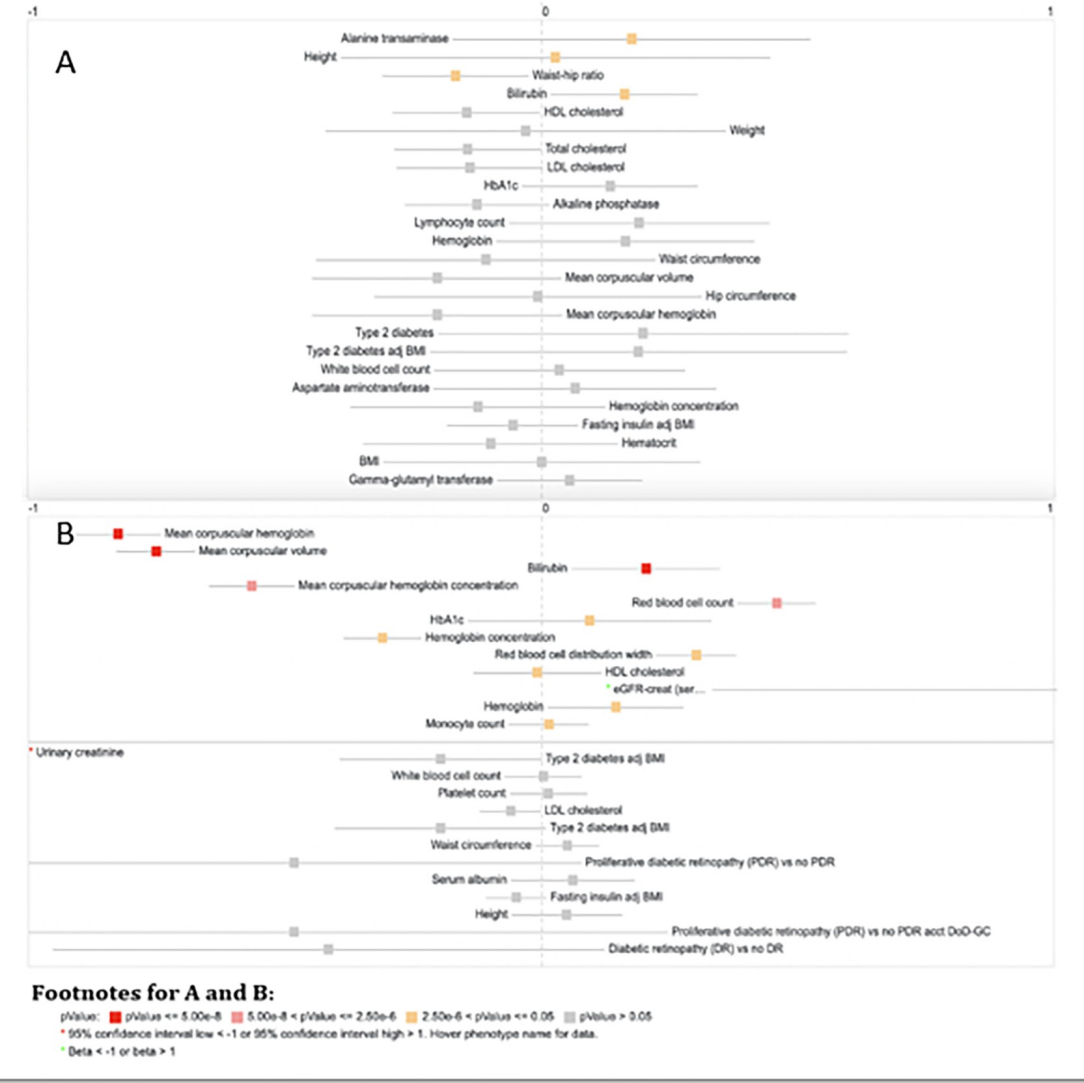
PheWAS forest plot of the top traits associated with the lead GWS loci in the UGR cohort (A) Chr 8 (rs374279268) and (B) Chr 16 (rs148110594).

Phenome-Wide Association Studies (PheWAS) were conducted using the lead SNPs from Table 2. rs374279268 and rs148110594. Fig 3 contains the summaries of the related PheWAS findings from the Open Target Genetics and the CMDKP portal datasets. From our PheWAS, several phenotypes were associated with loci from Chr 8 and Ch16, respectively, in Fig 3. For instance, prior GWS associations were seen for mean corpuscular hemoglobin, mean corpuscular volume and bilirubin.

## Discussion

This is the first multivariate GWAS analysis of liver biomarker levels in continental Africans. In this study, we investigated the genetic variants that were associated with liver biomarker levels in rural Ugandans, using a multivariate GWAS approach. SNPs rs374279268 and rs148110594 were significantly associated with liver function in the UGR cohort and were also found to be replicated in the SZC cohort.

*RHPN1* which is known as Rhophilin-Like Rho-GTPase Binding Protein 1 has not been previously associated with liver biomarker levels. A paralog of this gene *RHPN2* has been reported to be associated with alkaline ALP and serum GGT liver enzyme concentration in the European population [39]. Subsequently, *RGS11* is known as Regulator of G-Protein Signalling 11, which negatively regulates G protein signaling, and may modulate cardiovascular activities. Gurdasani *et al*, 2019, reported another SNP (rs151330263) related to this gene via the enhancer region to be associated with bilirubin levels [19]. This confirms our observations, as rs148110594 was found to be associated with liver biomarker levels in Ugandans, and the results replicated in Zulus. Interestingly, a similar gene family, *RGS6*, has been linked to ALP measurement in UK Biobank, which includes Europeans, Asians, and Africans [40], as well as *RGS6*, has also been linked to AST measurement in European and East Asian populations [17]. Based on NCBI’s Allele Frequency Aggregator (ALFA) pipeline, the African, East-Asian, and European reference allele frequencies of rs374279268 were 0.9941, 1.000, and 1.000 respectively. While that of rs148110594 were 0.9728, 1.0000, and 1.0000 respectively for African, East-Asian, and European. These two SNPs are monomorphic as they have the same allele across the genome.

The mvLMM analysis of the SZC shows a significant association to the UGT1A gene also known as UDP glucuronosyltransferase family 1 member A1. This gene encodes a UDP-glucuronosyltransferase, an enzyme of the glucuronidation pathway responsible for the metabolism of substances such as drugs and bile in the body. Studies by Chen et al has also reported the association of this gene to bilirubin level [37, 41].

Subsequently, our genetic findings were also supported by functional analyses for fundamental liver health and function processes from in silico pathway and gene set analysis in MAGMA to PheWAS. In particular, our loci were linked with Chr16p13, Chr 8q24, breast cancer amplicons, cellular components of the haptoglobin and hemoglobin complex, and the molecular functions of haptoglobin binding and oxygen carrier activities in our gene set analysis. Interestingly, our GWAS findings on the Nikolsky breast cancer 16p13 amplicon curated gene set was significant (Table 2), as well as the individual input genes in the gene set were enriched (Fig 2A & 2B). This is likely linked to the high fraction of effector proteins, including proteases and metabolic enzymes concentrated on 16p13 amplicons [42]. Furthermore, metastatic breast cancer studies have shown 50–70% of autopsied breast cancer cases developed liver metastasis [43] highlighting this genetic region may play an essential role in liver disease.

In addition, from our findings from the enrichment of input genes in gene sets for cellular components; the haptoglobin and hemoglobin complex, and related molecular functions in haptoglobin binding and oxygen carrier activity in Fig 2C–2E were enriched. Although no direct evidence from this present study, we hypothesise that this enrichment may be because haptoglobin is produced by the liver, which the body uses to clear free Hemoglobin (found outside of red blood cells) from circulation [44, 45]. Thus, highlighting this region may play an essential role in liver function, particularly, in Africans.

Subsequently, in other large cohorts, the PheWAS showed significant associations that were linked to plausible phenotypes, namely, mean corpuscular hemoglobin, mean corpuscular volume and bilirubin. These findings have promising functions from in silico pathway and gene set analyses for liver biomarker levels, as well as in other correlated phenotypes like red blood cell components and bilirubin. For example, the liver’s bilirubin is a yellowish substance made during the body’s normal process of breaking down red blood cells. Bilirubin is found in bile, a fluid in the liver that helps digest food. If the liver is healthy, it will remove most of the bilirubin from the body [46].

Our study’s first strength was it included the first and largest multivariate GWAS for six liver biomarker levels, which allowed us to discover SNPs associated with liver biomarker levels in continental Africans. This meant that the top loci were from correlated liver biomarker traits, and this improved the statistical power, which allowed us to find novel associations. Second, our study included functional analyses which helped explain our plausible findings, which were replicated in an urban South African Zulu population. Thus, our study was adequately powered to perform the first study of its nature in continental Africans for liver biomarker levels.

Our study’s limitation was that the UGR cohort, did not have the popular UDP glucuronosyltransferase family 1 member A1 (*UGT1A1)* locus (rs887829), which is known to be involved in the conjugation of bilirubin and associated with serum total bilirubin levels in the Chen *et al*., 2021 study [37, 41, 47]. However, the UGT1A1 locus was available and significant (P-value < 0.05) in the SZC cohort. Furthermore, other UGT1 family loci were also available in the SZC findings. We plan that future studies will explore the UDP glucuronosyltransferase family of genes further, and include the *UGT1A1* region, as it was reported to be common in all populations it was tested for in univariate liver biomarker levels, including in African Americans and West Africans [17, 41, 47]. Second, our tissue expression was not consistent nor conclusive in the UGR cohort, which may indicate the need for more African data for functional work and referencing. The utility of multivariate analysis in improving discovery was not fully achieved in this study as there were not so many differences between this study and Gurdasani’s et al., study where univariate analysis had been undertaken. Finally, the rural Ugandan sample reports many blood disorder related findings, however, our sample is from a region with a history of increased susceptibility to blood disorders, which may be confounding our results. Thus further, analyses are needed to better understand our findings.

## Conclusion

In conclusion, using the multivariate GWAS method improves power to detect novel genotype-phenotype associations for liver functions not found with the standard univariate GWAS in the same dataset. SNPs with a shared genetic basis associated with multivariate GWAS for six liver biomarker levels in rural Ugandans were replicated in urban South Africans. These results demonstrate the value of performing a multivariate GWAS in continental Africans with the opportunity to gain further insight into the genetic architecture of the liver. Larger studies and more functional work needs to be performed to further explore the genetic architecture of the liver in continental Africans.

## Supporting information

S1 File (XLSX)

## List of abbreviations

LFTs: Liver Function Tests
ALT: Alanine Transaminase
AST: Aspartate Transaminase
ALP: Alkaline Phosphatase
GGT: Gamma-Glutamyl Transferase
GWAS: Genome-Wide Association Study
LMM: Linear Mixed Model
QQ: Quantile-Quantile
GEMMA: Genome-wide Efficient Mixed-Model Association
UGR: Ugandan Genome Resource
SZC: South Africa Zulu cohort
GPC: General Population Cohort
DDS: Durban Diabetes Study
DCC: Diabetes Case Control
SNPs: Single Nucleotide Polymorphism
SST: Serum Separation Tube
LD: Linkage Disequilibrium
PheWAS: Phenome-Wide Association Studies
FUMA: Functional Mapping and Annotation
MAGMA: Multi-marker Analysis of GenoMic Annotation
MsigDB: Molecular Signatures Database
GO: Gene Ontology
